# The chemotherapeutic activity of areca nut extract increased stromal tumor-infiltrating lymphocytes in 4-nutriquinoline-1-oxide-tumor-induced Sprague-Dawley rats

**DOI:** 10.12688/f1000research.125784.1

**Published:** 2022-12-23

**Authors:** Liza Meutia Sari, Cut Fera Novita, Poppy Andriany, Dina Keumala Sari

**Affiliations:** 1 Oral Medicine, Universitas Syiah Kuala, Banda Aceh, Indonesia, 23111, Indonesia; 2 Public Dental Health, Universitas Syiah Kuala, Banda Aceh, Indonesia, 23111, Indonesia; 3 Tropical Disease and Nutrition, Universitas Sumatera Utara, Medan, Indonesia, 20155, Indonesia

**Keywords:** Areca nut, Oral cancer, Tumor-infiltrating lymphocytes, 4NQO-tumor inducer

## Abstract

**Background:** Oral squamous cell carcinoma (OSCC) is one of the most common oral cancers with a high mortality rate. The biodiversity source in Indonesia makes areca nut a potential antioxidant in treating disease.

**Objective:** The study aimed to evaluate the chemotherapeutic effect of areca nut extract in 4-nutriquinoline-1-oxide (NQO)-tumor-induced rats.

**Methods:** Twenty-eight male Sprague-Dawley rats were divided into four groups. Group 1 served as the control group, group 2 was 4NQO-induced rats without treatment, and groups 3 and 4 were given 4NQO-tumor inducer with 500 and 1000 mg/kg BW of areca nut extract, respectively. The rats in groups 2,3, and 4 received 30 ppm of 4NQO tumor inducer in drinking water for 12 weeks. In the end, all rats were euthanized and the tongue was removed. The body, liver, kidney, heart, and lungs weights were measured. Tongue tumor volume and dysplasia lesions were analyzed. The tumor-infiltrating lymphocytes (TILs) in the tumor and stromal area were scored semi-quantitatively associating the infiltrate grade (0-3) and analyzed histologically.

**Results:** There were significant differences in body weight loss between the initial and final phases in groups 1 and 2 (p<0.05). The areca nut at doses of 500 and 1000 mg/kg BW significantly reduced the tumor size compared with groups 1 and 2 (p<0.05). The incidence of OSCC in rats with 500 and 1000 mg/kg BW of areca nut extract after 22 weeks was 0%, but the dysplasia lesions were observed at 28.57% and 85.71%, respectively. The highest mean of stromal TILs was in group 3 and there were significant differences in stromal TILs between groups 2 and 3 (p<0.05).

**Conclusion:** Areca nut extract exerts a chemotherapeutic activity in 4NQO-induced rats by inducing infiltrating lymphocytes in the stromal tumor area on the OSCC lesion of the tongue.

## Introduction

Indonesia is a rich country in biodiversity including herbal plants. About 80% of the world's herbal plants growing spread across various islands in this country. Areca nut ( *Areca catechu* Linn.) is a nut that contains flavonoids and polyphenolics, especially arecoline, catechin, epicatechin, and quercetin has proved to show antioxidant activity which is beneficial for human health. ^1^ Areca nut is also one of substances that is frequently used by people after tobacco, alcohol, and caffeinated drinks according to WHO. ^2^ Nowadays, the habit of chewing the areca nut as part of the hereditary heritage and a source of encouragement to work is still used by people in several rural and mountain areas in Indonesia, especially in Aceh and North Sumatera, Indonesia. This chewing custom is also often consumed in India in the form of paan masala and gutkha. ^3^ Areca nut chewing is also one of the favorite habits in Taiwan. ^4^ Potency antioxidant activity of areca nut has been well established in the previous in vitro research using several oral cancer cell lines. ^5^ Although some areca nut products such as paan masala (fennel seeds, coriander, sesame, mint leaves, powdered lime, areca nut, cardamom, and pure menthol) and gutkha (chewing tobacco, dried ripe areca nut, paraffin wax, slaked lime (calcium hydroxide), and savory or sweet flavorings) have contribution in pathogenesis of oral squamous cell carcinoma (OSCC), several other studies have been carried out to prove the efficacy and safety of areca nuts depending on the type of phytochemical content possessed by the areca nut species from the certain region.

Oral squamous cell carcinoma is a cancer that most often causes a high mortality rate. ^6^ The clinical characteristic of OSCC in a certain population shows that smoking and drinking alcohol are the most frequent factors causing oral malignancies. Other etiology factors that can cause oscc are human papilloma virus (HPV), nutritional deficiency, candidal infection, and genetic predisposition. ^7^ The clinical appearance of oscc is ulcerated lesion with necrotic base in the central area surrounded with elevated border. In the last five years, many new cases of oscc and pharyngeal cancer appeared in all over the world and more than 7,000 cases caused death from these cancers. ^8^ The best current OSCC treatment still depends on surgery combined with chemo/radiotherapy. Although this is still the best option, this therapy has many side effects due to its non-selective activity including mucositis, osteoradionecrosis, xerostomia, dysgeusia, and orofacial pain so research to find alternative therapies derived from herbal ingredients is still being developed. ^9^ This herbal therapy is expected to inhibit the development of malignancy cells but has no destructive effect on healthy cells so that side effects of chemo/radiotherapy in the oral cavity can be minimized. Currently, there are no reports that examine whether the intake of areca nut extract is beneficial and safe for consumption in OSCC patients, considering the effects of OSCC induction by areca nut which are feared for humans when consumed in the long term. However, several studies have been carried out to develop the effects of areca nut on health. ^10^^,^^11^ The oral manifestations of OSCC are generally in the form of ulcerated lesions which are infiltrated by lymphocytes and inflammatory cells such as monocytes, macrophages, and plasma cells.

Recently, several new therapies have used reactive anticancer immune responses to cancer to treat breast cancer. ^12^ Tumor-infiltrating lymphocytes (TILs) located in the microenvironmental of the lesion site can predict tumor biology and the prognosis of cancer treatment. ^13^ Several clinical studies have also evaluated the importance of TILs as a determinant and predictive value in mammary cancer prognosis. ^14^^,^^15^ A previous study reported that the mean TILs for T lymphocytes were generally lower in OSCC patients who died compared to those who survived. ^16^ Another study has shown high lymphocytic infiltrate, especially infiltration of T and B lymphocytes in ductal carcinoma in situ. ^17^ Currently, a more in-depth research is needed to develop the efficacy of areca nut on the development of oral cancer. To support further *in vivo* research, an animal model is needed that uses the chemical carcinogen 4-nitroquinoline-1-oxide (4NQO) to form OSCC lesions. This 4NQO mouse model can induce OSCC with a similar progression step in OSCC and describes a carcinogenesis process similar to that of chronic tobacco abuse in humans. ^18^ In this study, we investigated the effect of areca nut extract on OSCC on the tongue using a mouse model given 4-NQO tumor inducer in drinking water. This study is expected to provide new information on the efficacy of areca nut in the progression of SCC on the tongue through clinical and histological examination.

## Methods

### Ethical clearance

This research was approved by the Ethical Clearance Committee Faculty of Dentistry, Universitas of Syiah Kuala, Banda Aceh, Indonesia, with the number 247/KE/FKG/2021. All research procedures were carried out based on the Organization for Economic Cooperation and Development (OECD), which indicated that all rats would receive human care based on the criteria contained in the section "Guide for the care and use of laboratory animals (OECD, 2015) and Institutional Animal Care and Use Commitee (IACUC) of this facility. All procedures in this study were reported following the ethical roles, principles, and guidelines of ‘Animals in Research: Reporting in vivo Experiments’ (ARRIVE). All the procedures have passed the ethic commitee and IACUC guidelines.

### Plant material

The areca nut is from an areca nut plantation in the province of Aceh Besar, Indonesia. Determination of plants in the form of roots, stems, leaves, and nuts, was identified by Dr. Sunaryo at the Herbarium Unit of the Botanical Division of Biological Research Center in Cibinong, West Java, Indonesia, with voucher specimen number 735/IPH.1.02/If.8/IV/2019.

### Aqueous extract of areca nut dose administration level

Two kilograms of ripe areca nut were washed under running water and dried in the sun and open air. The drying process was continued by using an electric oven at 50°C for 30 minutes for 5 days. The dried areca nut samples were crushed into a fine powder and then filtered using a 20-mesh sieve. The 1.7 kg of fine powder was soaked in 96% ethanol at 20-25°C for 7 days. The ethanol extract was then evaporated from the solvent so that it became a dry powder using a rotary evaporator at a temperature of 30-40°C. The dry powder was stored at room temperature. The aqueous extract of areca nut is made by mixing dry areca nut powder with water. The doses used in this study were 500 and 1000 mg/kg BW. This dose was chosen based on a safe or non-toxic dose after oral acute toxicity tests were carried out in our previous acute oral toxicity study. The areca nut extract solution was mixed with distilled water so that it had a liquid consistency and was easy to give to rats by giving it every day which was divided into 2 doses, morning and evening.

### Animals

A total of 28 male Sprague-Dawley rats that were pathogens free aged 8-10 weeks old, with a body weight of 180-250 g came from the Pharmacology Laboratory, Faculty of Veterinary Medicine, Universitas Syiah Kuala, Banda Aceh. Animals were put in animal cages with stainless steel grid cover (seven rats in a cage) in a stable environment and have free access to drinking water and food which is put in polypropylene bottles. Acclimatization of animals has carried out at a temperature of 25-30°C and with a 12 h light/dark cycle for a week before the experiments. We also feed dietary supplement to increase the stamina of the rats.

### Induction of oral carcinogenesis by 4-nitroquinoline-1-oxide

The anticarcinogenic test was carried out based on the guidelines of OECD guidelines for testing chemicals in animals (OECD, 2001). Oral squamous cell carcinoma induction was performed using 4-Nitroquinoline 1-oxide (4NQO Cas No. N8141-5G, Sigma Aldrich). This carcinogenic chemical agent was prepared by mixing 30 ppm 4NQO with drinking water and then given daily to rats in light-shielded water bottles for 12 weeks. ^19^ The bottle was protected from sun exposure to protect it from chemical changes that may affect the 4NQO’s efficacy. The drinking water was changed every week.

### Experimental procedure of chemotherapeutic study

This study was designed to observe the chemotherapeutic effect of areca nut extract on the healing of OSCC on the tongue induced using 4NQO in rats. A total of 28 male Sprague-Dawley rats were randomized into 4 groups. Groups 1 and 2 were control groups. Group 1 was untreated and group 2 was oral cancer induced by 4NQO administration without treatment. Groups 3 and 4 were induced by 30 ppm 4NQO administration and treated with areca nut extract at 500 and 1000 mg/kg body weight (BW), respectively. The areca nut extract treatment was started 1 week after cessation of the 4NQO administration. In this study, the observation of the health condition of the rats was done every day in the morning to identify possible deaths from toxicity from the 4NQO administration during the induction and treatment periods. The body weights were routinely measured every week and at the end of the experimental period. At the end of this experimental study, all rats were anesthetized through the intramuscular injection of 50 mg/kg BW ketamine 100 mg/mL and 5 mg/kg BW of xylazine 100 mg/mL. Prior to the injection stage, the rat’s cages were covered by black cover for 20-30 minutes to reduce stress in rats. The rats were sacrificed followed by excision of the whole tongue to assess the volume of tongue tumor analysis. The experimental protocol is shown in Table 1. The clinical examination also includes the calculation of the weight and condition of the heart, lung, liver, and kidney. The tongue of six rats in each group was longitudinally cut into halves for the histopathological examination. The tongue tissue was then fixed in 10% buffered formalin, embedded in paraffin blocks, and stained with hematoxylin and eosin (H & E).

**Table 1.** Research flow for administration of 4NQO and areca nut extract in Sprague-Dawley rats.

Groups	Treatment		
	1-12 weeks	1 week	20-22 weeks
Group 1 (Untreated control)			
Group 2 (OSCC induced without treatment)			
Group 3 (Treatment with 500 mg of areca nut)			
Group 4 (Treatment with 1000 mg of areca nut)			

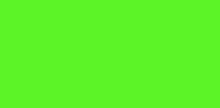
 Basal diet and tap water

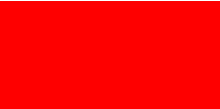
 4NQO 30 ppm in drinking water

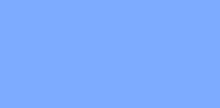
 500 mg of areca nut extract in diet

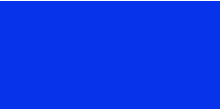
 1000 mg of areca nut extract in diet

### Tongue tumor volume analysis

Tongue tumor volumes were analyzed at the end of the experimental study and measured based on the formula length x width x height x π /6. ^20^ The tongue sections were analyzed histologically and classified as dysplasia, hyperplasia, and OSCC in every rat. ^21^

### Morphological evaluation of tumor-infiltrating lymphocytes

This study aims to evaluate TILs using a methodology for the TIL scoring system developed by Denkert *et al.* and the International TILs Working Group based on the H & E slides. ^22^^,^^23^ Observations were made on intratumoral and stromal TILs. Intratumoral TILs are lymphocytes in direct cell-to-cell contact with the cancer cells with no intervening stroma. ^24^ Stromal TILs are located clustered or scattered in the stroma among the cancer cells and do not directly interact with the cells. ^25^ The evaluations carried out in both TILs with the assessment of **score 0**: no infiltrating lymphocytes; **score 1**: mild increase of infiltrating lymphocytes in the tumor nest or stroma; **score 2**: increase infiltrating lymphocytes interwoven with tumor issue; **score 3**: prominent infiltrating lymphocytes separate or incorporated in tumor tissue. ^26^

### Histological examination

The buccal mucosa tissue was fixed in 10% formal saline for 24 hours, the tissue was dried or dehydrated using absolute ethyl alcohol, then embedded in paraffin at a temperature of 50°C. The wax tissue blocks were then sectioned by sliding microtome, stained with H & E, and analyzed under the binocular microscope (Olympus CX23 with an attached digital camera). Slides were subsequently reviewed by the pathologist.

### Statistical analysis

The data were analyzed using the SPSS version 20. The quantitative results were expressed as the mean ± SD. Body weight was analyzed by using a dependent t-test. Data representing heart and lungs weights, tumor volume, intratumoral, and stromal TILs, were compared using the non-parametric Kruskal-Wallis test with post hoc comparisons made using the Mann-Whitney test to determine the significance of the differences between groups. The liver and kidney weights were compared by using a one-way ANOVA test. The results were statistically significant at a *p*-value <0.05. The data of incidence of tongue dysplasia were displayed in term of percentage.

## Results

### Body weight analysis

During the research, from the first week of the adaptation period until the end of the treatment, no rats died. The mean weight changes of rats were seen to increase and decrease significantly in the control and Ca-induced control group (Group 1 and 2) when compared before and after treatment for 22 weeks. There were no significant weight changes in other groups ( Table 2). Although groups 3 and 4 were Ca-induced with areca nut extract treatment, there was no weight loss observed after the experimental study.

**Table 2.** Effect of areca nut extract on body weight changes.

Groups	Body weight changes (g)			
	Initial	Final	95% CI	*p*
**Group 1**	207.67 ± 18.18	219.00 ± 23.68	-18.53 to -4.133	0.010
**Group 2**	208.00 ± 8.60	195.00 ± 12.24	5.43 to 20.56	0.007
**Group 3**	208.33 ± 8.69	211.33 ± 12.11	-7.09 to 1.09	0.118
**Group 4**	208.83 ± 8.49	211.50 ± 11.89	-6.95 to 1.62	0.170

Data are expressed as the mean ± SD (n= 28), 95% CI, *p*<0.05, dependent t-test. Group 1: untreated control; Group 2: Ca-induced control; Group 3: treatment with 500 mg/kg BW of areca nut extract; Group 4: treatment with 1000 mg/kg BW of areca nut extract.

### Heart, lungs, liver, and kidney weight analysis

The study showed that there were no significant differences in heart, lungs, liver, and kidney weights among the groups ( Table 3). The heart and lung weights in the 4NQO-induced rat groups appeared to be lighter than in the normal group although this difference was not significant. Liver and kidney weights in groups 2, 3, and 4 were heavier than the normal group.

**Table 3.** Effect of areca nut extract on heart and lungs, liver, and kidney.

	Weight (g)				
	Group 1	Group 2	Group 3	Group 4	*p*
**Heart and lungs**	5.95 ± 0.92	4.71 ± 2.12	4.39 ± 0.85	4.98 ± 1.13	0.057
**Liver**	10.38 ± 2.06	10.53 ± 1.39	10.77 ± 1.11	9.73 ± 1.53	0.638
**Kidney**	2.36 ± 0.42	2.47 ± 0.77	2.37 ± 0.26	2.31 ± 0.69	0.957

Data are expressed as the mean ± SD (n= 28), *p*<0.05. Heart and lung weight analysis using Kruskal Wallis test. Liver and kidney weight analysis using ANOVA test. Group 1: untreated control; Group 2: Ca-induced control; Group 3: treatment with 500 mg/kg BW of areca nut extract; Group 4: treatment with 1000 mg/kg BW of areca nut extract.

### Tongue tumor volume analysis

The tumor tissue began to appear on the tongue and can be observed macroscopically in the 22 weeks of the experimental period. As shown in Table 4, the treated group with 500 and 1000 mg/kg BW of areca nut extract showed a smaller lesion compared with group 1 (untreated group) and group 2 (untreated and induced by 4-NQO group).

**Table 4.** Effect of areca nut extract on tongue tumor volume.

	Group 1	Group 2	Group 3	Group 4	*p*
**Tongue tumor volume (mm3)**	0.00 ± 0.00 ^a^	14.86 ± 3.66 ^b^	0.15 ± 0.11 ^a^ ^,^ ^b^	0.16 ± 0.05 ^a^ ^,^ ^b^	0.000

Data are expressed as the mean ± SD (n= 28), *p*<0.05. p-values were derived when tongue tumor volume results for each group were statistically compared using the Kruskal-Wallis test and Mann-Whitney post hoc test. Group 1: untreated control; Group 2: Ca-induced control; Group 3: treatment with 500 mg/kg BW of areca nut extract; Group 4: treatment with 1000 mg/kg BW of areca nut extract.

^a^ significantly different for Group 1 vs. Group 2, 3, 4.

^b^ significantly different for Group 2 vs. Group 3, 4.

### Incidence of tongue dysplasia

During the induction of tumors on the rat's tongue, dysplasia and OSCC lesions occurred on the dorsolateral and posterior surfaces of the tongue. The incidence of tongue dysplasia after treatment with areca nut extract is shown in Table 5. The incidence of OSCC in rats after being treated with areca nut extract at 500 and 1000 mg/kg BW was 0%, but the dysplasia lesions can be observed at 28.57% and 85.71%, respectively. The incidence of hyperplasia lesions in the 500 mg/kg BW treatment group was observed at 71.43%. The specimen that has been diagnosed as having OSCC on a certain area of the tongue, the dysplasia and hyperplasia areas could also still be detected in a few parts of the same lesion.

**Table 5.** Incidence of hyperplasia, dysplasia, and OSCC lesions after treatment.

Group	Number of rats observed	Number of rats (%)		
		Hyperplasia	Dysplasia	OSCC
**Group 1**	7 (100%)	0 (0.00%)	0 (0.00%)	0 (0.00%)
**Group 2**	7 (100%)	0 (0.00%)	2 (28.57%)	5 (71.43%)
**Group 3**	7 (100%)	5 (71.43%)	2 (28.57%)	0 (0.00%)
**Group 4**	7 (100%)	1 (14.29%)	6 (85.71%)	0 (0.00%)

Data are expressed as the percentage of the number of rats (n=28), *p*<0.05. Group 1: untreated control; Group 2: Ca-induced control; Group 3: treatment with 500 mg/kg BW of areca nut extract; Group 4: treatment with 1000 mg/kg BW of areca nut extract.

### Intratumoral and Stromal TILs Analysis

The results of the univariate analysis showed that the mean score of intratumoral lymphocyte infiltration in 4NQO-induced rats that were given areca nut extract orally at doses of 1000 mg/kg BW every day showed the lowest average intratumoral TILs among the groups. Meanwhile, the normal and 500 mg/kg BW areca nut extract had the highest mean TILs of 0.83 or with a mild increase of infiltrating lymphocytes in the tumor nest. The study did not find significant differences in the mean of intratumoral TILs in all groups ( *p*>0.05) as shown in Table 6. In a subsequent test performed on stromal TILs, it was observed that the highest mean of stromal TILs was in group 3 (4NQO induced rats with 500 mg/kg BW of areca nut treatment) ( Table 6). This study showed a significant difference in stromal TILs between group 2 (4NQO induced rats without treatment) and group 3 ( *p*<0.05).

**Table 6.** Effect of areca nut extract on intratumoral and stromal TILs.

	Group 1	Group 2	Group 3	Group 4	*p*
**Intratumoral TILs**	0.83 ± 0.41	0.50 ± 0.54	0.83 ± 0.41	0.33 ± 0.55	0.411
**Stromal TILs**	0.83 ± 0.41	0.50 ± 0.84 ^a^	1.67 ± 0.52 ^a^	1.17 ± 0.41	0.023

Data are expressed as the mean ± SD (n= 24), *p*<0.05. p-values were derived when intra and stromal TILs results for each group were statistically compared using the Kruskal-Wallis test and Mann-Whitney post hoc test. Group 1: untreated control; Group 2: Ca-induced control; Group 3: treatment with 500 mg/kg BW of areca nut extract; Group 4: treatment with 1000 mg/kg BW of areca nut extract.

^a^ significantly different for Group 2 vs. Group 3.

### Histopathological analysis

**Figure 1.**
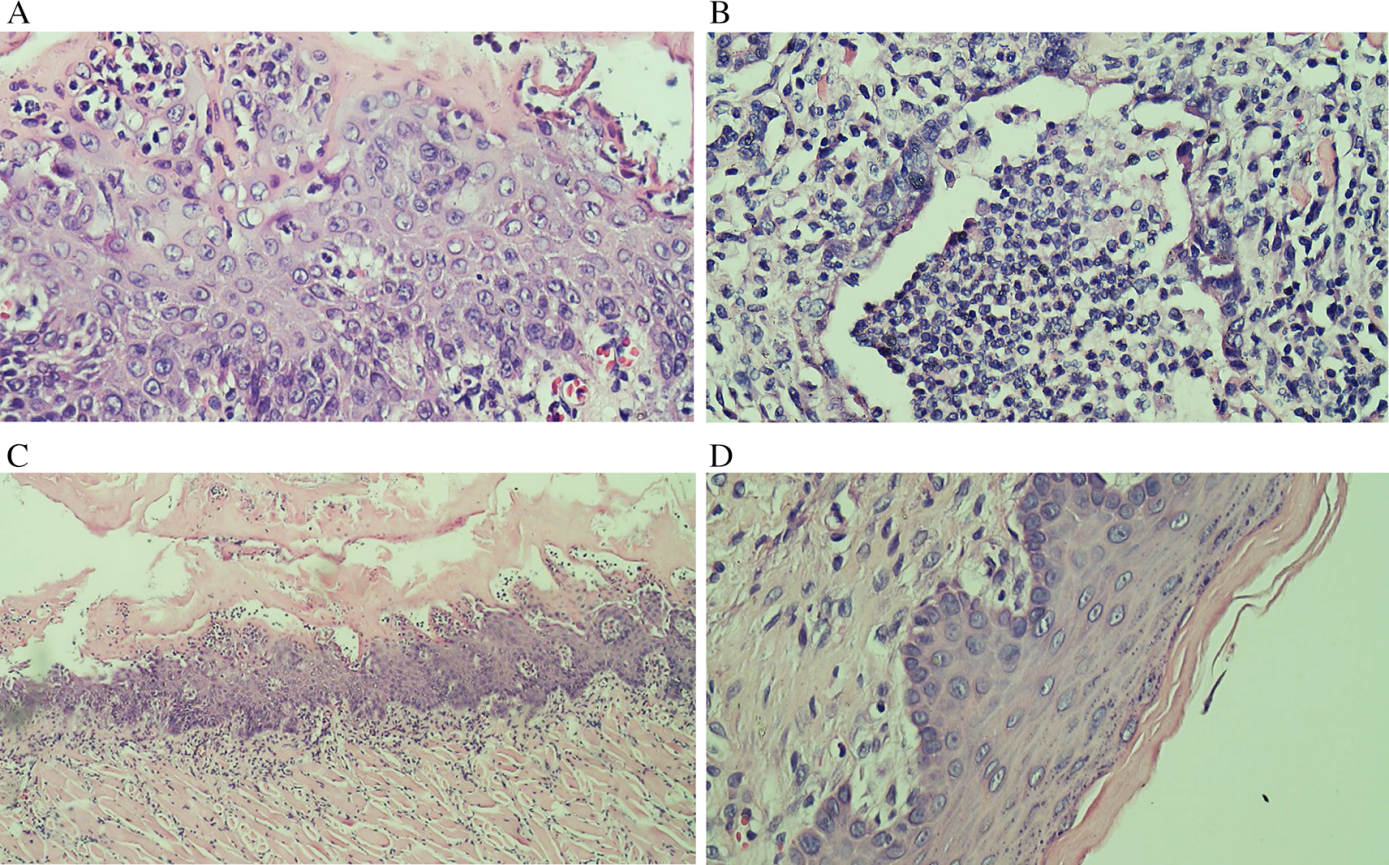
The level of intratumoral TILs of tongue stained with H & E staining (400x). (A) The low level of TILs showed the mild increase of infiltrating lymphocytes in the normal tongue; (B) The TILs score 2 in the Ca-induced control group; (C) The score 1 of TILs in tongue treated with 500 mg/kg BW areca nut extract (100x); (D) The score 0 of TILs in tongue treated with 1000 mg/kg BW areca nut extract.

**Figure 2.**
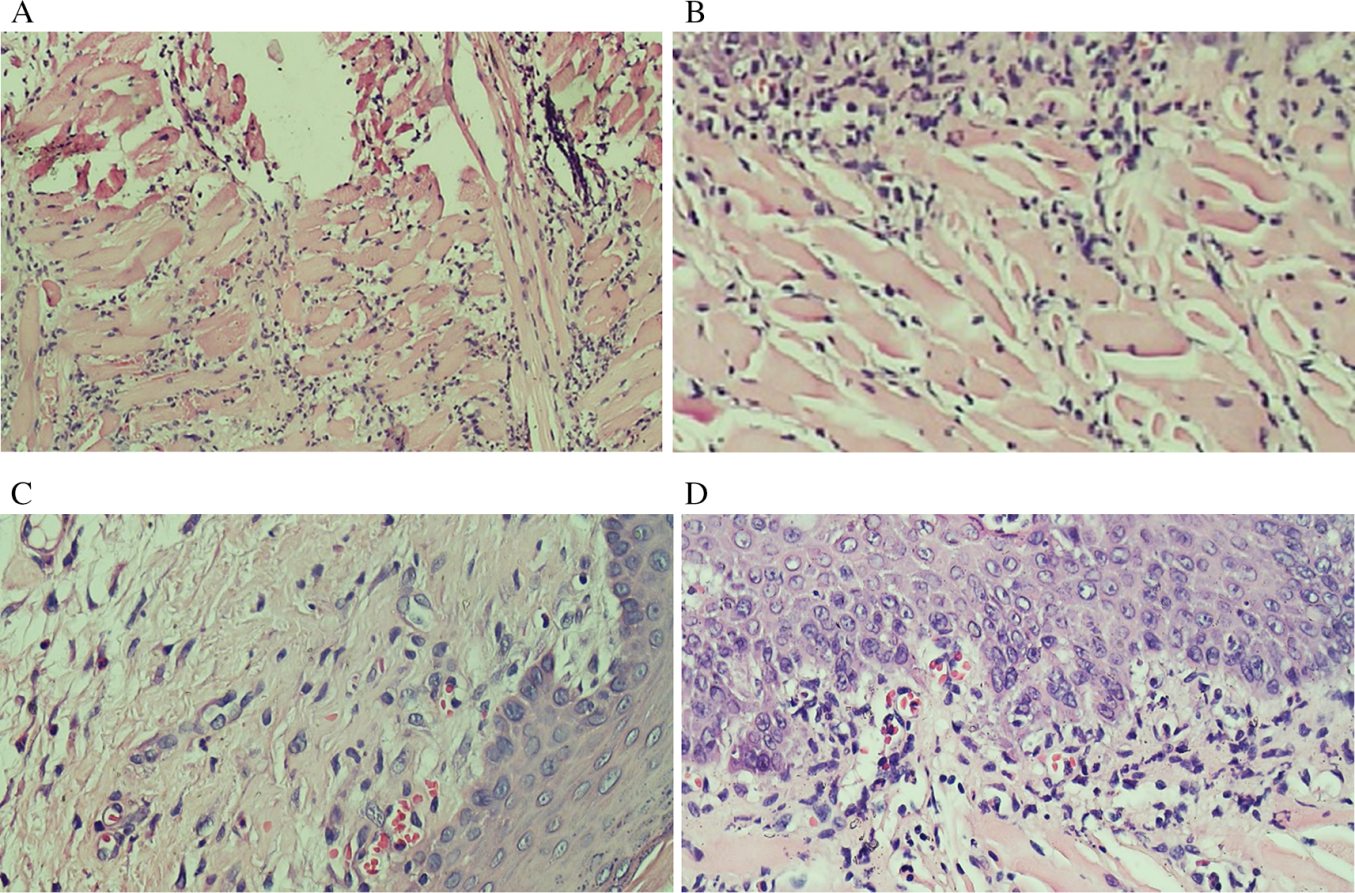
The level of stromal tumoral TILs of tongue stained with H & E staining (400x). (A) The TIL score 1 in the normal tongue; (B) The TIL score 2 in the Ca-induced control group; (C) The TIL score 1 in tongue treated with 500 mg/kg BW areca nut extract; (D) The score 1 of TILs in tongue treated with 1000 mg/kg BW areca nut extract (100x).

Evaluation results of the final histological feature were performed using light microscopy by qualified pathologists. The histological appearance of intratumoral TILs of each group can be seen in Figure 1. Accumulation of inflammatory cells especially polymorphonuclear cells or intratumoral TILs 1 was seen in Figure 1B. The TILs 0 in rats treated with areca nut extract 1000 mg/kg BW is observed in Figure 1D which showed the stratified squamous cell epithelium without inflammatory and tumor cells. The basal layer was intact in the specimen. The histological feature of stromal TILs can be seen in Figure 2. The stromal TILs showed an accumulation of lymphocytes that spread in the stroma area and were not directly in contact with the cancer cells.

## Discussion

The *in vivo* assay using an animal model is an important part of investigating the development of new therapy for the disease, especially those from new herbal medicine sources. Currently, the development of herbal plants as oral cancer treatment is still being explored. Up to now, the use of areca nut extract for OSCC treatment has not been reported. This study tried to reveal the role of areca nut extract in aqueous as a chemotherapeutic in OSCC induced by 4NQO-tumor inducer in Sprague-Dawley rats. The experimental research was designed by using randomization in a subject grouping. No rats died in this study. The rats were relatively stable in uniformity in age, sex, body weight, and acclimatization process.

The current study used 30 ppm 4NQO for 12 weeks followed by chemotherapy treatment for up to 22 weeks. This result is in line with a previous study that reported that administration of 30 ppm 4NQO in drinking water for 4-9 weeks and 36-46 weeks caused OSCC in 25% and 75%, respectively. ^27^ Another study reported that with 20 ppm 4NQO for 10 weeks, the OSCC will experience reproducible power as much as 83% at 26 weeks of administration. ^28^ The choice of 4NQO in drinking water is a trigger for dysplastic and neoplastic lesions because it can trigger tumors that morphologically and histopathologically have similarities to human tumors. The length of time the appearance of cancerous lesions on the tongue varies greatly depending on the period of time of administration and dose of the inducer. In addition to the appearance of oral lesions, we found several potential systemic toxicities in experimental animals including discoloration of the lungs, liver, and kidneys, and darkening of the skin accompanied by weight loss. Based on a previous study, some of the changes that occurred due to the administration of 4NQO were focal hepatic lobular necrosis, renal tubular degeneration, decreased cellularity in the splenic, reduction of body weight and white blood cell count, and increased of liver serum. ^29^

We found there was significant weight loss occurred in 4NQO-induced rats without treatment. The decrease in body weight is generally caused by a loss of appetite, eating disorder, and an increase in metabolic rate because of the OSCC lesion in the oral cavity. ^30^ Interestingly, this study also revealed that there were increases in body weight after treatment with the areca nut, although they were not significant. This is probably due to the areca nut extract’s ability to increase body weight. Its mechanism of action is that the main component of areca alkaloids is an inhibitor of the gamma-aminobutyric acid (GABA) receptor. ^31^ The inhibitory effects of GABA receptor may increase appetite with eventual fat accumulation in general and central area of the body. Another mechanism that might play role in gain weight is that the areca nitrosamines derived from alkaloids may induce insulin resistance which can cause hyperglycemic conditions. Another study has reported that areca nut chewing has closely associated with metabolic syndrome, general obesity, and type-2 diabetes mellitus. ^32^

The 4NQO is a synthetic carcinogen derivative of quinoline, sensitive to light, easily affected by temperature changes, and soluble in water. The mechanism of action of the 4NQO-tumor inducer is that it can induce the activation of COX-2 which will release nitric oxide (NO), especially inducible nitric oxide synthase (iNOS) which induces intracellular oxidative stress. ^33^ Its action begins with an enzymatic reduction of the nitro group and then produces its carcinogenic potential by producing additional DNA to form hydroxydeoxyguanosine (8OHdG) that can cause oxidative damage. ^34^ The damage caused by 4NQO is similar to the lesions caused by other carcinogenic substances such as alcohol and tobacco. The morphological and histopathological changes also have similarities with OSCC in humans.

Our results showed that treatment of areca nut extract affected the reduction of tongue tumor size and incidence of tumor dysplasia. This study is a chemotherapeutic study which means that the areca nut may affect the progression phase of carcinogenesis. Areca nut has a high level of polyphenols and flavonoids such as catechin, tannin, and quercetin that act as antioxidants. We did not find much research regarding the anticancer activity of areca nut extract, but many studies have explored the effect of catechin, one of the most abundant phytochemicals contained in the areca nut on carcinogenesis in vitro and in vivo. Xu *et al.* reported that catechin might inhibit the tobacco-specific nitrosamines induced tumorigenesis through inhibition of the 8OhdG. Another research by Yoshimura *et al.* reported that catechin especially epigallocatechin gallate can inhibit cancer cell proliferation by inducing apoptosis and inhibiting cell cycle progression. ^35^ Catechin can induce p53-mediated cell death through stabilization and activity of p53, inhibit the release of TNF-α which is a proinflammatory cytokine present in cancer, inhibit proliferation by causing cell cycle arrest in the G0/G1 phase, modulate apoptosis-related signaling, diminish mitochondrial membrane potential, neutralize ROS, and inhibit angiogenesis. ^36^ Arecoline is also the primary phytochemical compound in areca nuts. Arecoline is recognized to cause oral submucosal fibrosis because it contains toxic constituents. This phytochemical content might be an obstacle to healing from oral cancer. Areca nut does not contain carcinogenic ingredients, but the toxic effect comes from nitrosamine products which are produced by the nitrosation process by dry arecoline which is chewed and digested by stomach acid for a long time and uncontrollable. Our research uses the areca nut extract for a certain period, so the possibility of areca nut extract causing cell damage such as fibrosis or pathogenesis of OSCC is quite small. In the crude extract that contained many phytochemical properties, there might be a mutually enhancing effect or vice versa from different chemical interactions in the crude extract. The size of extract volume does not determine the efficacy of phytochemical compounds that produce the extract's antioxidant effect. Fractionation of the extract plant might be one of the solutions to get the original antioxidant content of the extract. This study is in line with the research of Al-koshab *et al.* that reported that *Ficus deltoidea* extract could significantly reduce the tongue tumor volume of OSCC at a high dose of 500 mg/kg BW and decreased the expression of key tumor marker cyclin D1 and increased β-catenin and e-cadherin antibodies. ^30^ Another study conducted by Roy M *et al.* uses black tea extract to prevent carcinogenesis by down-regulating protein tyrosine kinases, reducing ROS level in blood, and resolving DNA damage. ^37^

The immune system is the main actor in the fight against the pathogenesis of many types of cancer, including OSCC. These complex defense mechanisms involve many varieties of lymphocytes. For OSCC, TIL might be a potential prognostic factor for determining the possibility of success of the treatment. Tumor-infiltrating lymphocytes are a heterogeneous collection of lymphocytes dominated by T cells. These cells generally appear in the stromal area and contain cytotoxicity activity against cancer cells. Caruntu *et al.* reported that TIL CD8 ^+^ lymphocytes and CD56 ^+^ in the intratumoral compartment can be predictive indicators of OSCC and they are associated with improved patient outcomes. ^38^ This study could not find a significant difference in intratumoral TILs among groups, but it showed an increase in the level of stromal TILs at 500 mg of areca nut extract treatment. Previous study showed that stromal TILs was found to be better and more reproducible parameters than intratumoral TILs. ^39^ The intratumoral TILs generally present in small numbers and mostly are detected in fewer cases of OSCC, more heterogeneous, and difficult to be observed on H & E-stained slides. Counting stromal TILs is more advantageous because stromal TILs have a clearer density and pattern of lymphocytes in carcinoma. Lymphocytes that are counted are only those in the spaces between carcinoma nests. In the microenvironment of a tumor, TILs may reflect tumor biology and predict the outcome of the treatment. Another study demonstrated that infiltrates of CD4 ^+^, CD8 ^+^, and FoxP3 ^+^ lymphocytes were associated with patient survival and can be potential biomarkers for OSCC. ^40^

Although this study has shown that the areca nut extract has a chemotherapeutic effect on 4NQO-induced rats in reducing tongue tumor volume and increasing the number of stromal TILs, there are several limitations to this study. First, this was a chemotherapeutic study that only investigated the efficacy of areca nut extract after tumor formation, while its function as chemopreventive study has not been explored especially in determining the antioxidant effect to prevent carcinogenesis in the initiation phase. Second, we did not explore the type of lymphocyte cells and tumor markers involved in the cytotoxicity mechanism of areca nut extract so we could not determine the exact mechanism of the areca nut extract in this chemotherapeutic activity. A more in-depth future experimental assay is needed to develop the results of this study.

## Conclusion

In conclusion, we have demonstrated that areca nut extract exerts a chemotherapeutic effect 4 NQO-induced rat tongue when fed during the progression phase of carcinogenesis by increasing the number of infiltrating lymphocytes in the stromal tumor area. Additional works are needed to investigate the chemopreventive effect and the exact molecular pathway involved in the anticancer activity of areca nut extract.

## Data Availability

Dryad: Underlying data for ‘The Chemotherapeutic Activity of Areca Nut Extract Increased Stromal Tumor-Infiltrating Lymphocytes In 4-Nutriquinoline-1-Oxide-Tumor-Induced Sprague-Dawley Rats. https://doi.org/10.5061/dryad.mkkwh713d The project contains the following underlying data: body, heart, lungs, liver, and kidney weights, tongue tumor volume, incidence of tongue dysplasia, intratumoral and stromal TILS, and Author checklist-E10 ARRIVE.
