# Supplementary material for: Areca nut extract increased stromal tumor-infiltrating lymphocytes in 4-nitroquinoline-1-oxide-tumor-induced Sprague-Dawley rats
Source: F1000Res. 2025 Jun 20;11:1571. Originally published 2022 Dec 23. [Version 4] doi: 10.12688/f1000research.125784.4 (PMC12184624; doi:10.12688/f1000research.125784.4)
Supplement: Peer review history

# Reviewer report: Reviewer response for version 3

**DOI:** 10.5256/f1000research.161333.r257814

**Published:** 2024-04-11

**Authors:** Jiaxuan Qiu

**Affiliations:** 1 Nanchang University, Nanchang, Jiangxi, China

This article mainly introduces the potential  therapeutic effects of  Areca nut on OSCC which is innovative.

 The paper is comprehensive ,but there are still shortcomings that need to be revised:

1. Where is the macroscopic view of tumors? A treatment comparison chart is required.
2. Table 4 is far from enough and P value can not be described as 0.000. The authors need to supplement datas.
3. As the author says that the body weights of rats were measured weekly ,then the graph of the weekly weights change should be made instead of a table comparing the initial and final weights.
4. What is the meaning of “score 2: increase infiltrating lymphocytes interwoven with tumor issue”? Maybe it is a spelling or Typographical error.
5. H&E appears to be too few for TIL validation, and immunohistochemistry or fluorescence experiments should be added to verify T cell infiltration.

Is the work clearly and accurately presented and does it cite the current literature?

Partly

If applicable, is the statistical analysis and its interpretation appropriate?

Partly

Are all the source data underlying the results available to ensure full reproducibility?

Partly

Is the study design appropriate and is the work technically sound?

Partly

Are the conclusions drawn adequately supported by the results?

Partly

Are sufficient details of methods and analysis provided to allow replication by others?

Yes

Reviewer Expertise:

Tumor microenvironment，Precision chemotherapy

I confirm that I have read this submission and believe that I have an appropriate level of expertise to confirm that it is of an acceptable scientific standard, however I have significant reservations, as outlined above.

# Reviewer report: Reviewer response for version 2

**DOI:** 10.5256/f1000research.145116.r188533

**Published:** 2023-08-09

**Authors:** Popovici Violeta

**Affiliations:** 1 Ovidius University of Constanta, Ilarie Voronca Street, Constanta, Romania

Forty-three references, only 14 from the last 5 years.

 Please, check and correct reference number 13.

 The reviewer suggests the title revision without "the chemotherapeutic activity." The authors could only show the areca nut extract action in the present tense.

 1. Introduction:

 The reviewer suggests beginning with the general data about OSCC and updating this section with the newest National Cancer Institute information regarding oral cancer therapy. Moreover, the authors could enumerate more causal factors of OSCC.

 After, they can link with herbal therapy and continue with the areca nut presentation. 

 The authors are encouraged to support their statements with more than 1 reference and to organize the data from this section better.

 2. Methods:

 The Plant material subsection could be changed in Plant extract, showing the areca nut extract preparation at the same time. 

 The administered doses of areca nut extract could be shown lately in the experimental procedure. At Statistical Analysis, the authors should indicate the year and provider of the software version.

 3. Results:

 The table footer should contain the each group signification to understand the data from tables better. 

 The same indication is available for the figures, with all abbreviations explained. 

 In the tables, the compared data should be better highlighted. 

 4. More recently published references are requested for Discussion. 

 5. The authors are encouraged to check and reformulate the Conclusion for better understanding.

Is the work clearly and accurately presented and does it cite the current literature?

Partly

If applicable, is the statistical analysis and its interpretation appropriate?

Partly

Are all the source data underlying the results available to ensure full reproducibility?

Partly

Is the study design appropriate and is the work technically sound?

Yes

Are the conclusions drawn adequately supported by the results?

Partly

Are sufficient details of methods and analysis provided to allow replication by others?

Yes

Reviewer Expertise:

Dental medicine, oral cancer, in vitro studies, microbiology, and immunology

I confirm that I have read this submission and believe that I have an appropriate level of expertise to confirm that it is of an acceptable scientific standard, however I have significant reservations, as outlined above.

# Response

**Published:** 2024-01-12

**Authors:** Liza Sari

**Affiliations:** Dentistry Faculty Syiah Kuala Indonesia, Indonesia

Dear Reviewer,

 Thank you very much for the thorough corrections.

 I apologize for the delay in making revisions because I am in the process of obtaining a functional position at the university so some journals are recommended to be postponed to publish completely until the process is complete. I hope this delay is understandable

**Review**: Forty-three references, only 14 from the last 5 years.

***Revision:** We have replaced 15 references to old journals with the latest journals that were published less than the last 5 years.*

**Review**: Please, check and correct reference number 13.

***Revision:** Thank you, We have corrected the mistake by changing the journal with the latest journal*

**Review**: The reviewer suggests the title revision without "the chemotherapeutic activity." The authors could only show the areca nut extract action in the present tense.

***Revision**: We have deleted the “Chemotherapeutic activity” in the title and draft manuscript.*

**Review**: The reviewer suggests beginning with the general data about OSCC and updating this section with the newest National Cancer Institute information regarding oral cancer therapy. Moreover, the authors could enumerate more causal factors of OSCC.

 After, they can link with herbal therapy and continue with the areca nut presentation. 

 The authors are encouraged to support their statements with more than 1 reference and to organize the data from this section better.

***Revision: **The changes have been made as instructed **. **We have added the data from the National Cancer Institute, the International Agency for Research on Cancer GLOBOCAN 2020, and Cancer Stat Facts*

**Review:** The Plant material subsection could be changed in Plant extract, showing the areca nut extract preparation at the same time. 

 The administered doses of areca nut extract could be shown lately in the experimental procedure. At Statistical Analysis, the authors should indicate the year and provider of the software version.

***Revision**: We have revised the subsection title and the software provider.*

**Review: **The table footer should contain each group's signification to understand the data from tables better. The same indication is available for the figures, with all abbreviations explained. In the tables, the compared data should be better highlighted.

***Revision:** We have made all the revisions in the results section.*

**Review**: More recently published references are requested for Discussion. 

**Revisions:** The addition of several journals

# Reviewer report: Reviewer response for version 2

**DOI:** 10.5256/f1000research.145116.r167035

**Published:** 2023-04-04

**Authors:** Maizatul Hasyima Omar

**Affiliations:** 1 Herbal Medicine Research Centre, Institute for Medical Research, National Institutes of Health, Ministry of Health, Malaysia, Shah Alam, Malaysia

I am satisfied with the author's responses to my questions/issues raised in my initial review. The revised manuscript can be accepted for indexing.

Is the work clearly and accurately presented and does it cite the current literature?

Yes

If applicable, is the statistical analysis and its interpretation appropriate?

Partly

Are all the source data underlying the results available to ensure full reproducibility?

Partly

Is the study design appropriate and is the work technically sound?

Yes

Are the conclusions drawn adequately supported by the results?

Partly

Are sufficient details of methods and analysis provided to allow replication by others?

Partly

Reviewer Expertise:

Phytochemistry

I confirm that I have read this submission and believe that I have an appropriate level of expertise to confirm that it is of an acceptable scientific standard.

# Reviewer report: Reviewer response for version 1

**DOI:** 10.5256/f1000research.138126.r159547

**Published:** 2023-02-02

**Authors:** Maizatul Hasyima Omar

**Affiliations:** 1 Herbal Medicine Research Centre, Institute for Medical Research, National Institutes of Health, Ministry of Health, Malaysia, Shah Alam, Malaysia

Overall, the manuscript was logically organized with a good flow among the introduction, methods, results, discussion, and conclusion. However, it will be good if the authors could write in more detail in terms of the procedure for animal study, i.e. the exact duration (week/day). The authors also failed to explain the reason for a one week gap in all groups (Table 1). For Table 2, the Initial and Final should refer to the week or day which is more appropriate. For Table 6 the value for the mean is referring to what unit? i.e. 0.83 for Group 1.

 It was reported that areca nut extract may caused oral cancer (Ji *et al.*, 2014 ^1^; Wadia, 2022 ^2^), how should the authors respond to these with their findings in vivo?

Is the work clearly and accurately presented and does it cite the current literature?

Yes

If applicable, is the statistical analysis and its interpretation appropriate?

Partly

Are all the source data underlying the results available to ensure full reproducibility?

Partly

Is the study design appropriate and is the work technically sound?

Yes

Are the conclusions drawn adequately supported by the results?

Partly

Are sufficient details of methods and analysis provided to allow replication by others?

Partly

Reviewer Expertise:

Phytochemistry

I confirm that I have read this submission and believe that I have an appropriate level of expertise to confirm that it is of an acceptable scientific standard, however I have significant reservations, as outlined above.

## References

[1] : Areca nut extracts exert different effects in oral cancer cells depending on serum concentration: A clue to the various oral alterations in betel quid chewers. *Toxicol Rep* .2014;1: 10.1016/j.toxrep.2014.10.018 1087-1095 10.1016/j.toxrep.2014.10.018 28962320 PMC5598531

[2] : Areca nut and oral cancer. *British Dental Journal* .2022;232(12) : 10.1038/s41415-022-4425-1 10.1038/s41415-022-4425-1 35750828

# Response

**Published:** 2023-03-03

**Authors:** Liza Sari

**Affiliations:** Dentistry Faculty Syiah Kuala Indonesia, Indonesia

Dear Reviewer, Thank you very much for the detailed correction. I would like to answer the correction that I have made in this article.

- The procedure took 36 weeks starting with 1 week for the acclimatization period, 12 weeks for 4NQO-tumor induction, and 1 week for the observation period after the 4NQO-tumor induction. We continued the experiment with 22 weeks for treatment with the areca nut extract period. We have added this statement in the “ **Discussion” section, **1 ^st ^paragraph.
- The observation period which is one week after the 4NQO-tumor induction aims to observe whether there was death or not in rats after being induced by 4NQO-tumor induction. This observation is done to anticipate if there was a dying rat that might happen in the early stage or right before the treatment experiment began. This period is also intended to prepare the rat’s condition before treatment, such as observing the activity, toxicity, consciousness, skin color, and body weight condition. After a week of observation, we continued with by 22 weeks treatment period with areca nut extract. We have added this statement in the “ **Discussion” section.**
- Initial body weight is the weight calculated on the last day of the 1 week observation period after the 4NQO-tumor induction period was complete. Meanwhile, the final body weight is the weight calculated after 22 weeks of the treatment with areca nut. We have added this statement in the “ **Body weight analysis”** section.
- This study adopted and modified the TIL scoring system used in previous studies. Firstly, the tumor area was divided into the center of the tumor and the invasive margin according to the criterion in the study of Galon et al. The invasive margin was defined as the junctional area between the tumor-invading edge area and the host stroma. The TIL evaluation was conducted operating in the feature of TIL intensity in the center and the invasive margin of the tumor was incorporated. TILs in each tumor on the tissue were manually using a 20x objective lens. The mean count of the scores for each subject was recorded and used in the analysis. TIL assessment was done by two pathologists who were blinded to the clinical appearance of the tumor. We have added this statement in the “ **Morphological evaluation of tumor-infiltrating lymphocytes” section.**

References:

 1.            Galon J, Mlecnik B, Bindea G, Angell HK, Berger A, et al. Towards the introduction of the 'Immunoscore' in the classification of malignant tumors. J Pathol 2014;232:199–209.

 2.            Zhang D, He W, Wu C, Tan Y, He Y, et al. Scoring System for Tumor-Infiltrating Lymphocytes and Its Prognostic Value for Gastric Cancer. Front. Immunol. 2019;10(71):1-11.

 Several differences may cause different results between Ji WT's article and this article:

1. Article written by Ji WT et al. using lyophilization or freeze-drying in extracting areca nut. This method is one of the drying methods to produce a stable powder product. In our study, we did not use this method.  We use the maceration technique which has the advantage of being able to retain the phytochemical compounds present in the areca nut. This technique used 95% ethanol and dried again by using a rotary evaporator until we had a solid powder of areca nut. The extract has also been tested for antioxidant activity DPPH with a value of 15.95 µg/mL. This result indicates that areca nut extract has good antioxidant activity. The extract also contains total flavonoids 238,5 mg CE/g, and total phenolic 80.3 mg TAE/g. We also found that there was a high level of catechin and quercetin in the extract (HPLC assay). We concluded that different types of extraction methods and areca nut origin types might give different results in areca nut’s cellular or antioxidant activities in various research.
2. We have also done the experimental regarding the cytotoxicity and apoptosis activity of areca nut in HSC-2, HSC-3, and Hacat cell lines. The results showed good results regarding the capability of areca nut in inducing apoptosis of oral cancer cell lines. It has been published in https://f1000research.com/articles/7-723 and https://scholar.ui.ac.id/en/publications/potential-antioxidant-and-cytotoxic-activities-of-areca-nut-areca .
3. We agree with Wadia R’s article about areca nut and oral cancer. Please let us give our opinion. There are a few reasons why areca nut can cause oral cancer, but in my article, areca nut has anticancer activity. Areca nut does not contain carcinogenic substances,  but this carcinogenic effect is caused by nitrosamines product in the long term and is uncontrollable.  Nitrosamines are produced by nitrosation of the alkaloids in dried stored nuts, when in the mouth, and especially in the acid conditions of the stomach, in the presence of nitric oxide generated by bacteria.  The combination with nitric oxide,  produced by bacteria,  causes the production of methylnitrosaminoproprionitrile  which is proven to cause carcinogenesis in animal studies.  This endogen nitrosation is higher significantly in a patient with bad oral hygiene.  If areca nut is combined with tobacco,  then chewed by people with bad oral hygiene,  there will be a very high accumulation of nitrosamines products.  This process usually occurs continuously for the long term because the seeds have addictive properties. Some studies also reported increased reactive oxygen species such as hydroxyl oxide in the oral cavity caused by a combination of polyphenol autooxidation from areca nut with high alkaline pH of slaked lime  (calcium hydroxide paste).  If the areca nut is chewed with piper betel and slaked lime,  these two materials will cause mucous membrane erosion so that a carcinogenic substance could easily penetrate cells through the mucous membrane.  Some part of communities in  India and  Pakistan use industrial packaging areca nuts called gutka and pan masala. Gutka contains areca nut, piper betel,  tobacco,  and slaked lime,  while pan masala was prepared without tobacco.  Approximately,  40%  of gutka and pan masala packaging are contaminated with aflatoxin which has carcinogenic properties from  Aspergillus flavus, Aspergillus niger, and Rhizopus spp.  fungi.  The occurrence of the  OSCC's risk depends greatly on the composition of the compound which determines the quality of the seeds, the method of using seeds which are associated with oral hygiene,  duration of use,  the presence or absence of toxins caused by fungi contamination in the seeds,  and the presence or absence of other carcinogenic substances such as tobacco and slaked lime.  The occurrence of OSCC could also be influenced by several factors such as intrinsic factors (tumor suppressor gene abnormality or mutation and oncogene)  and extrinsic factors  (tobacco smoking,  vitamin  A and iron deficiency, candidiasis, viral infection, and immunosuppression).
